# Patient Satisfaction With Surgery of Basal Cell Carcinoma: Keep It Safe and Simple

**DOI:** 10.7759/cureus.76891

**Published:** 2025-01-04

**Authors:** Roxana Behnejad, Raphael Herr, Eleni Koliakou, Axel Zahn, Wiebke K Peitsch

**Affiliations:** 1 Department of Dermatology and Phlebology, Vivantes Klinikum im Friedrichshain, Berlin, DEU; 2 Faculty of Medicine, Charité University Medicine, Berlin, DEU; 3 Department of Medical Informatics, Biometry and Epidemiology, Professorship of Epidemiology and Public Health, Friedrich-Alexander-Universität Erlangen-Nürnberg (FAU), Erlangen, DEU; 4 Dermatology, Hautärzte Dr. Linder, Frankfurt, DEU

**Keywords:** basal cell carcinoma, rotation flap, satisfaction, skin graft, surgery, wound closure

## Abstract

Background: Patient-reported outcomes are important quality indicators in dermatologic surgery.

Objectives:We performed a prospective cohort study to assess patient satisfaction with surgery of basal cell carcinoma (BCC) and identify patient-, tumor-, and treatment-related influencing factors.

Methods: Patients who underwent BCC surgery at the Vivantes Skin Cancer Center in Berlin, Germany, rated global, cosmetic, and functional satisfaction on a visual analog scale from 0 (very dissatisfied) to 10 (very satisfied) at suture removal (T0) and three months after surgery (T1). Group differences were examined with the Kruskal-Wallis test and independent associations with linear regressions.

Results: Among 150 participants (41.3% females, mean age 75.3 years), 82.7% had a BCC in the head/neck region. Reconstruction after micrographically controlled excision was performed most frequently with advancement or rotation flaps (52%), followed by linear closure (27.3%), transposition flaps (8.7%), and skin grafts (6%). Patient satisfaction was overall high (global: mean 8.5 (T0) and 8.3 (T1), cosmetic: 7.3 (T0) and 7.6 (T1), functional: 8.6 (T0) and 8.2 (T1)) and intercorrelated with physicians’ satisfaction. Males and patients aged >75 years were significantly more satisfied with cosmetic and functional results. Poor health state, tumor localization on the nose, complications, pain, and prolonged wound healing were associated with lower satisfaction in most categories. Linear wound closure led to better global satisfaction than local flaps and skin grafts at T1.

Conclusion:Patient satisfaction was related to inherent patient- and tumor-associated factors, reconstruction technique, and complications, aspects to be discussed during shared decision-making to reconcile patients’ expectations with practicability.

## Introduction

Basal cell carcinoma (BCC) is the most common (semi)malignant tumor in white-skinned individuals worldwide [1,2]. The incidence continues to rise, with >200 new cases per 100,000 inhabitants per year in Germany and even >700 new cases in Australia [3-7]. The lifetime risk of developing BCC is estimated at 30% in the white-skinned population [8]. BCC occurs predominantly in the elderly (mean age 72 years) [5,7,9]. However, recently, there has also been an increase in younger patients, especially in women [10,11]. The tumor is preferentially localized in sun-exposed areas, particularly on the head and neck [4,8,12]. It is characterized by infiltrative and locally destructive growth and may cause extensive tissue damage in cosmetically critical areas, while metastasis is very rare (0.0028-0.55%) [10,13]. Provided that surgery is a viable option, complete excision is considered the first-line therapy for many types of BCC [8,14,15]. BCC in non-critical localizations may be excised with safety margins followed by conventional histological examination [8,14,15]. However, BCC with a high risk of recurrence, recurrent BCC, and BCC in critical localizations should be treated with micrographically controlled surgery, which employs a step-by-step, tissue-conserving surgical technique and a systematic evaluation of the resection margins [8,13,14,16]. Depending on not only the size and localization of the BCC and the elasticity of the surrounding tissue but also on patient characteristics, health state, and preferences, reconstruction can be achieved with primary linear closure, flaps, or skin grafts. Another option suitable for some cases and tumor localizations is secondary intention healing.

Treatment goals of patients and physicians include optimal functional and aesthetical outcomes, a low rate of recurrence, and the lowest possible risk of complications. In a discrete choice experiment on patient preferences for the treatment of BCC by Martin et al., a low recurrence rate was considered as most important, followed by cosmetic outcome [17]. Preferences were significantly influenced by patient- and tumor-associated factors, and cosmetic outcome was more important for patients with BCC on the head or neck [17].

As part of patient-centered care, patient satisfaction with the treatment of BCC is an important quality indicator, and information on influencing factors is crucial for optimal patient counseling [18-20]. Even if BCC is the most common skin cancer worldwide, evidence of patient satisfaction with BCC surgery and different reconstruction techniques is limited, and there are only a few prospective studies with this focus [18,21-24]. The aim of our prospective cohort study was to examine the treatment satisfaction of patients who underwent BCC surgery in our skin cancer center between 2018 and 2020 at the time of suture removal and three months after surgery and to identify patient-, tumor-, and treatment-related impact factors on global, cosmetic, and functional satisfaction.

## Materials and methods

Study participants

Patients were recruited from the Department of Dermatology and Phlebology, Vivantes Klinikum im Friedrichshain, Berlin, Germany, between December 1, 2018, and November 30, 2020. Inclusion criteria were histologically confirmed BCC, age ≥18 years, ability to provide written informed consent, and German language skills. The study was approved by the Ethics Committee of the Faculty of Medicine of Charité University Medicine Berlin (EA4/098/17) and conducted according to the principles of the Declaration of Helsinki.

Data collection

All patients who had given written informed consent were asked about treatment satisfaction on the day of suture removal during a routine visit to the clinic (T0) and three months after surgery by mail (T1).

The survey at T0 contained questions on gender, age, marital status, school degree, professional qualification, working status, comorbidity (documented by physicians), anticoagulation (documented by physicians), previous skin cancer surgery, number of excised skin cancers, tumor recurrence, kind of anesthesia, and complications (see Appendix A for the links to the questionnaire - both the German and English language versions). The patient-perceived general health state was documented on a visual analog scale (VAS; 0 = very poor, 10 = very good). Similarly, VAS was used to record global satisfaction with surgery, satisfaction with cosmetic and functional outcomes (0 = very dissatisfied, 10 = very satisfied), patient-perceived severity of complications, and postoperative pain (0 = not severe, 10 = very severe). Patients with multiple BCC were asked to rate treatment satisfaction for the predominantly treated, most bothersome tumor.

At the same time (T0), a physician questionnaire containing information on the number, anatomical localization, and histological subtype of the BCC, the number of operations, the type of wound closure, comorbidities, anticoagulation, complications and satisfaction with the cosmetic and functional outcome from the physician’s perspective on a VAS from 0 to 10 was completed for each participant by RB, who had not performed any of the operations.

At T1, study participants received a second questionnaire (see Appendix A for the links of the questionnaire - both the German and English language versions) and a prepaid envelope for return by mail, in which the general health state and satisfaction in all categories were reassessed after three months on a VAS. In addition, participants were asked for the duration of wound healing, persistent complications, and pain.

Statistical analysis

For descriptive analyses, percentages or mean values were calculated. For subgroup analyses, the participants were stratified according to gender, age (≤75 versus (vs.) >75 years), marital status (partnership vs. single/divorced/widowed), school degree (low, intermediate or high), professional qualification (apprenticeship or university degree), number of current skin cancers (1 or ≥2), tumor localization (head/neck or body; nose or other parts of the face), histological subtype (nodular vs. superficial vs. infiltrating BCC), number of comorbidities (≤3 or >3), anticoagulation, history of skin cancer, and general health state (<7 or ≥7 on a VAS from 0 to 10). Subgroup analyses were also performed for the type of wound closure (linear closure, advancement or rotation flap, transposition flap, or skin graft), physician-confirmed complications (yes or no) and duration of wound healing (≤2, 2-6, or >6 weeks). Differences in treatment satisfaction between groups were tested for significance by the non-parametric Kruskal-Wallis test. Associations between the number of comorbidities as a linear variable and patients’ satisfaction and between patients’ and physicians’ satisfaction with the cosmetic and functional outcome at T0 were examined with Spearman’s correlation.

The independent association of patient, disease, and treatment characteristics with global, cosmetic, and functional satisfaction at T0 and T1 was investigated by multivariate linear regression analyses. Separate models were calculated for each satisfaction category, with satisfaction as the dependent variable (transformed to reach normal distribution) and gender, age, general health state, number of comorbidities, professional qualification, marital status, tumor localization, reconstruction technique, complications, pain, and duration of wound healing as independent variables. Significance was assumed for p-values <0.05.

## Results

Patient and disease characteristics

A total of 179 patients with clinically suspected and/or histologically confirmed BCC were invited to participate. Ten patients refused to participate due to lack of time, and 19 had to be retrospectively excluded because the clinically suspected diagnosis of BCC was not confirmed by histopathology. Hence, data from 150 participants were included in the final analysis; among them, 41.3% (n = 62) were females (Table 1). The mean age was 75.3 years, 65.7% (n = 85) were living with a partner, and 34.7% (n = 52) had a university degree. Approximately half (54%, n = 81) suffered from more than three comorbidities (Table 1; for details, see Appendix B), and 46% (n = 69) rated their general health state as <7 on a VAS from 0 to 10. Noteworthy, 44.0% (n = 66) reported intake of anticoagulation (Table 1; for details see Appendix C). In addition, 51.3% (n = 77) of the patients had a history of previous skin cancer, and 33.3% (n = 50) presented with more than one BCC. The total number of skin tumors that were surgically removed simultaneously was n = 226, among which 73.5% (n = 166) were in the head/neck region. The study participants were asked to indicate their treatment satisfaction with the predominantly treated, most bothersome BCC (n = 150), which was located in the head/neck region in 82.7% (n = 124) of the cases and on the nose in 22% (n = 33; Table 1).

**Table 1:** Patient and tumor characteristics. ^a^ Percentages do not always sum up to 100% due to rounding and single missing values (total cohort: n = 150). ^b^ Homemaker or unemployed. ^c^ Measured on a visual analog scale (VAS) from 0 (very poor) to 10 (very good), 7 = median. ^d^ For details, see Appendix B. ^e^ For details, see Appendix C. ^f^ In the subgroup of patients with more than one BCC, the tumor localization and the histological subtype are indicated for the predominantly treated, most bothersome BCC which was taken as the basis for assessment of treatment satisfaction. ^g^ The total number of skin tumors surgically removed simultaneously in the study cohort was n = 226. The vast majority of these tumors were BCC. However, in a few patients with more than one skin tumor histopathology revealed precancerous skin lesions such as Bowen’s disease or advanced actinic keratosis or benign skin tumors such as irritated seborrheic keratosis in addition to the predominantly treated BCC for which treatment satisfaction was examined. Patients who had cutaneous squamous cell carcinoma, aggressive non-melanoma skin cancer (e.g., Merkel cell carcinoma), or malignant melanoma in addition to a BCC were excluded from the study. BCC: basal cell carcinoma

Characteristics	n (%)^a^
Age, mean (range)	75 (32-98)
Gender	
Female	62 (41.3%)
Male	88 (58.7%)
Marital status	
Partner	85 (65.7%)
Single/divorced/widowed	65 (43.3%)
School degree	
Low	42 (28.0%)
Intermediate	44 (29.3%)
High	55 (36.7%)
Education	
Apprenticeship	87 (58.0%)
University degree	52 (34.7%)
Working status	
Working	15 (10.0%)
Retired	133 (88.7%)
Not working^b^	2 (1.3%)
General health state, VAS 0-10^c^	
≥7	79 (52.7%)
<7	69 (46.0%)
>3 comorbidities^d^	
Yes	81 (54.0%)
No	69 (46.0%)
Anticoagulation^e^	66 (44.0%)
Previous skin cancer	77 (51.3%)
Tumor localization^f^	
Head/neck	124 (82.7%)
Nose	33 (22.0%)
Face, other than nose	79 (52.7%)
Other localization	26 (17.3%)
Recurrent BCC	11 (7.3%)
Number of treated BCC	
1	100 (66.7%)
≥2	50 (33.3%)
Total number of surgically removed skin tumors^g^	
1	100 (66.7)
2	33 (22.0%)
3	11 (7.3%)
4	4 (2.7%)
5	1 (0.7%)
6	1 (0.7%)
Histological subtype^f^	
Nodular	91 (60.7%)
Superficial	28 (18.7%)
Infiltrating	26 (17.3%)

Treatment characteristics

A majority (88.7%, n = 133) of the participants had single-stage micrographically controlled surgery, mostly in local anesthesia (82.7%, n = 124; Table 2). Regarding the reconstruction technique, advancement or rotation flaps were used most commonly in 52% (n = 78) of the defects, followed by linear closure after ellipse excision in 27.3% (n = 41), whereas transposition flaps (8.7%, n = 13) and skin grafts (6%, n = 9) were performed less frequently (Table 2). Histopathology revealed that 94.7% (n = 142) of the BCC were removed R0 with sufficient distance to the healthy tissue. Among all participants, 32.9% (n = 49) reported postoperative pain, and 18.7% (n = 28) suffered from any kind of complication, protracted or disturbed wound healing (Table 2; for details, see Appendix D). Wound healing including resolution of small residual crusts was completed within ≤2 weeks in 7.3% (n = 11), within >2-6 weeks in 52% (n = 78), and after >6 weeks in 23.3% (n = 35) of the participants.

**Table 2:** Treatment characteristics. ^a^ Percentages do not always sum up to 100% due to rounding and single missing values (total cohort: n = 150). ^b^ The subgroup with another type of wound closure included n = 2 patients who received a V-to-T flap on the cheek or on the temple, n = 2 patients with an S flap on the cheek or on the temple, n = 2 patients with secondary intention healing on the scalp or in the nasal canthus, and n = 1 patient each with a W-to-Y flap on the upper lip, an H flap on the forehead, and a wedge excision on the ear. ^c^ Any kind of complications or wound healing disturbance (for details, see Appendix D).

Characteristics	n (%)^a^
Anesthesia	
Local	124 (82.7%)
Standby or analgosedation	6 (4.0%)
General	20 (13.3%)
Re-excision due to R1 situation	8 (5.3%)
Type of wound closure	
Linear closure	41 (27.3%)
Advancement or rotation flap	78 (52.0%)
Transposition flap	13 (8.7%)
Skin graft	9 (6.0%)
Other^b^	9 (6.0%)
Duration of wound healing	
≤2 weeks	11 (7.3%)
>2-6 weeks	78 (52.0%)
>6 weeks	35 (23.3%)
Complications^c^	28 (18.7%)

Treatment satisfaction

**Figure 1:**
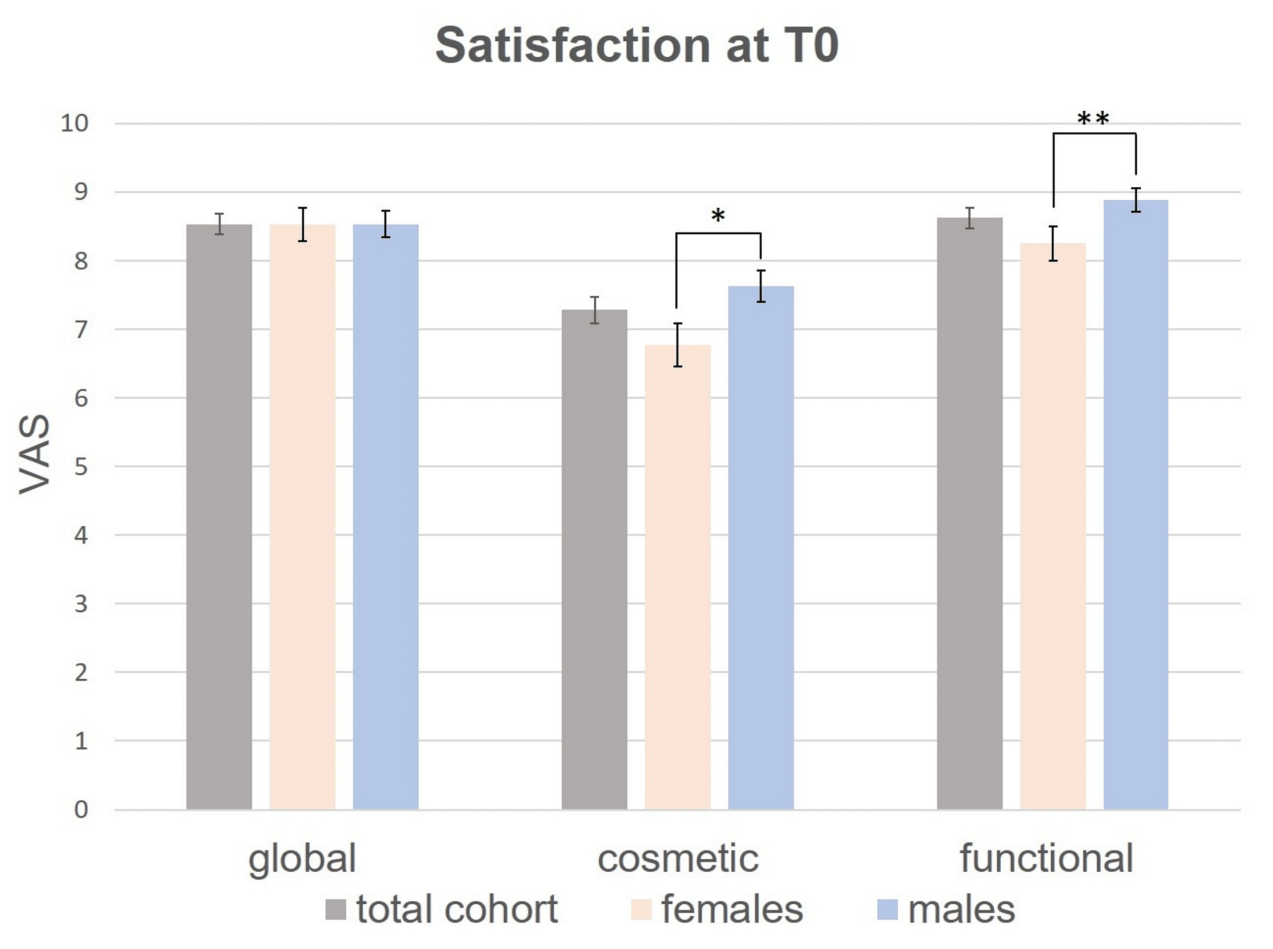
Mean satisfaction of the total study cohort, females and males on a visual analog scale (VAS) from 0 to 10 at T0. Differences in treatment satisfaction between groups were tested for significance by the non-parametric Kruskal-Wallis test. Bars: standard errors. * p <0.05, ** p <0.01. Exact p-values are indicated in Table 3.

At the time of suture removal (T0), the mean global patient satisfaction score was 8.5, the mean satisfaction with the cosmetic result was 7.3, and the mean satisfaction with functional outcome was 8.6 (n = 150 respondents; Fig. 1).

**Figure 2:**
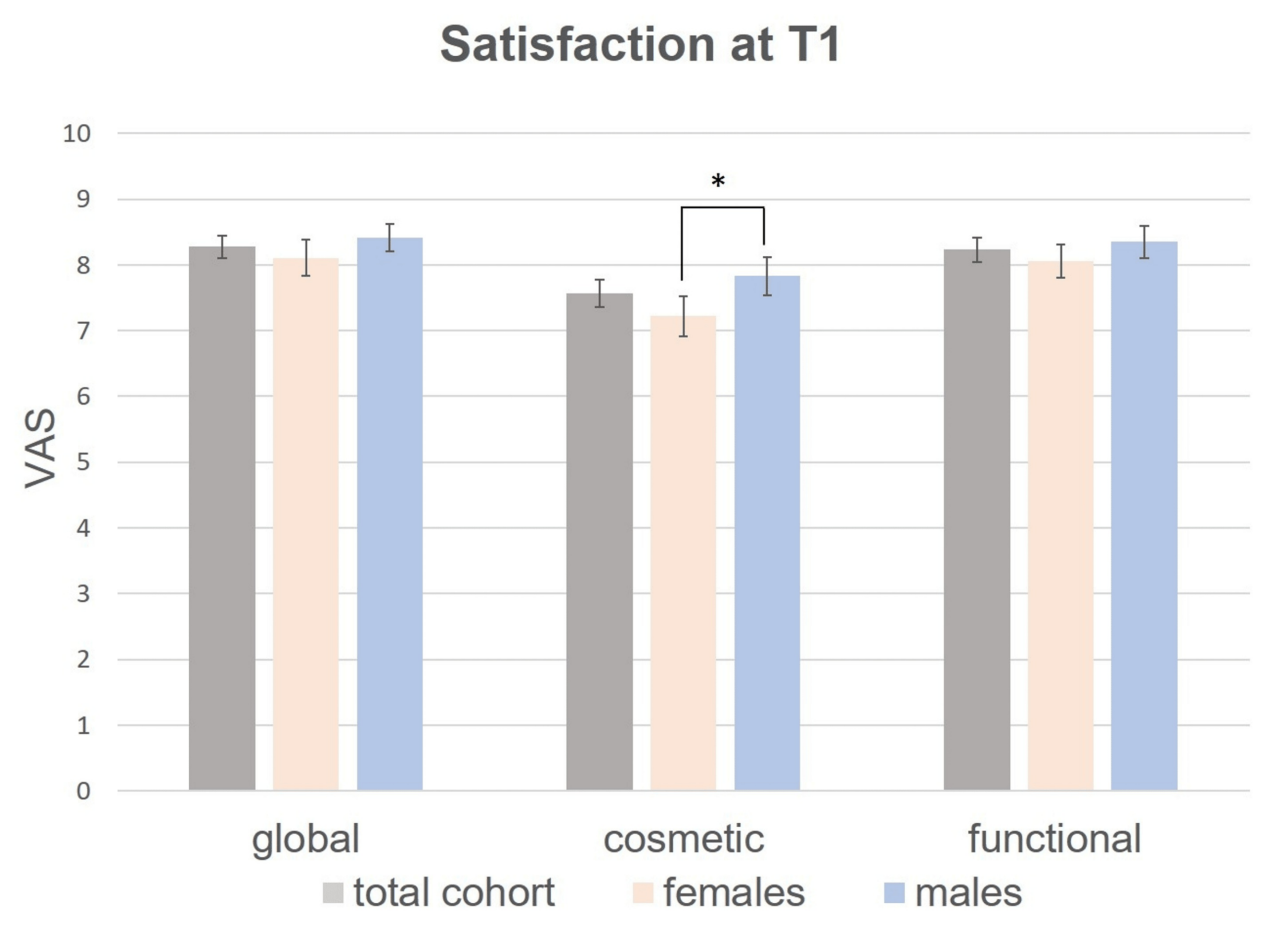
Mean satisfaction of the total study cohort, females and males on a visual analog scale (VAS) from 0 to 10 at T1. Differences in treatment satisfaction between groups were tested for significance by the non-parametric Kruskal-Wallis test. Bars: standard errors. * p <0.05. Exact p-values are indicated in Table 4.

After three months (T1), the patients reported similar scores with a mean global satisfaction of 8.3, a mean cosmetic satisfaction of 7.6, and a mean functional satisfaction of 8.2 (n = 127 respondents; Fig. 2).

Males were significantly more satisfied with the cosmetic outcome than females both at T0 (Fig. 1, Table 3) and at T1 (Fig. 2, Table 4). Higher cosmetic satisfaction of male participants at T0 was confirmed in multivariate regression models adjusted for confounders (ß = -0.21 for females vs. males, p = 0.027; Table 5). Furthermore, males were significantly more content with the functional outcome than females at T0 (Fig. 1, Table 3) but not at T1 (Fig. 2, Table 4).

**Table 3:** Patients’ satisfaction dependent on sociodemographic, disease, and treatment characteristics at T0. VAS: visual analog scale from 0 (very dissatisfied) to 10 (very satisfied), indicated as mean. ^a^ Measured on a VAS from 0 (very poor) to 10 (very good), 7 = median. ^b^ For patients with more than one BCC, the tumor localization, histological subtype, and kind of wound closure are indicated for the predominantly treated, most bothersome BCC that was taken as a basis for the assessment of treatment satisfaction. ^c^ The reference category for nose was other parts of the face. ^d^ Patients who received other types of wound closure including V-to-T flap (n = 2), S flap (n = 2), W-to-Y flap (n = 1), H flap (n = 1), wedge excision on the ear (n = 1), or secondary intention healing (n = 2) were not considered in this subgroup analysis due to the small number of cases. Differences in treatment satisfaction between groups were tested for significance by the non-parametric Kruskal-Wallis test. Significant p-values are highlighted in bold.

Characteristics	Global satisfaction			Cosmetic satisfaction		Functional satisfaction	
(n = 150)	VAS	p		VAS	p	VAS	p
Gender							
Female	8.53	0.841		6.77	0.033	8.25	0.008
Male	8.53			7.63		8.88	
Age							
≤75 years	8.51	0.625		7.33	0.863	8.66	0.948
>75 years	8.55			7.25		8.60	
Partner							
Yes	8.52		0.922	7.57	0.136	8.72	0.538
No	8.54			6.88		8.49	
Education							
Apprenticeship	8.29		0.082	7.02	0.202	8.26	0.013
University degree	8.80			7.45		9.15	
General health state^a^							
≥7	9.09		<0.001	7.59	0.129	9.01	0.009
<7	7.93			6.98		8.22	
Comorbidities							
≤3	8.85		0.047	7.49	0.431	8.79	0.500
>3	8.26			7.11		8.48	
Tumor localization^b^							
Head/neck	8.52		0.936	7.35	0.308	8.65	0.919
Other body parts	8.6			6.97		8.50	
Nose^c^	7.81		0.005	6.48	0.009	8.56	0.258
Face, other parts	8.86			7.62		8.77	
Number of treated BCC							
1	8.61		0.395	7.47	0.170	8.66	0.806
≥2	8.38			6.90		8.55	
Histological subtype^b^							
Nodular	8.55		0.866	7.36	0.702	8.73	0.295
Superficial	8.76			7.51		8.79	
Infiltrating	8.30			6.90		8.05	
Wound closure^b,d^							
Linear closure	8.60		0.499	7.26	0.930	8.74	0.686
Advancement or rotation flap	8.55			7.20		8.52	
Transposition flap	8.18			7.53		8.36	
Skin graft	8.65			7.83		9.08	
Complications							
Yes	7.55		<0.001	5.60	<0.001	7.71	0.015
No	8.76			7.63		8.82	
Pain							
Yes	7.98		0.007	6.34	0.002	7.85	0.003
No	8.80			7.72		9.00	

**Table 4:** Patients’ satisfaction at T1. VAS: visual analog scale from 0 (very dissatisfied) to 10 (very satisfied), indicated as mean. ^a^ Measured on a VAS from 0 (very poor) to 10 (very good), 7 = median. ^b^ For patients with more than one BCC, the tumor localization, histological subtype, and kind of wound closure are reported for the predominantly treated, most bothersome BCC that was taken as a basis for the assessment of treatment satisfaction. ^c^ The reference category for nose was other parts of the face. ^d^ Patients who received other types of wound closure including V-to-T flap (n = 2), S flap (n = 2), W-to-Y flap (n = 1), H flap (n = 1), wedge excision on the ear (n = 1) or secondary intention healing (n = 2) were not considered in this subgroup analysis due to the small number of cases. Differences in treatment satisfaction between groups were tested for significance by the non-parametric Kruskal-Wallis test. Significant p-values are highlighted in bold.

Characteristics	Global satisfaction			Cosmetic satisfaction		Functional satisfaction	
(n = 127)	VAS		p	VAS	p	VAS	p
Gender							
Female	8.11		0.480	7.22	0.043	8.06	0.113
Male	8.41			7.83		8.35	
Age							
≤75 years	8.18		0.658	7.35	0.192	7.78	0.045
>75 years	8.33			7.69		8.49	
Partner							
Yes	8.32		0.832	7.59	0.729	8.51	0.022
No	8.22			7.53		7.84	
Education							
Apprenticeship	8.33		0.818	7.50	0.708	8.00	0.068
University degree	8.40			7.65		8.52	
General health state^a^							
≥7	8.68		0.002	7.80	0.147	8.65	0.013
<7	7.90			7.30		7.84	
Comorbidities							
≤3	8.63		0.098	7.98	0.121	8.36	0.265
>3	7.94			7.17		8.11	
Tumor location^b^							
Head/neck	8.37		0.052	7.59	0.308	8.19	0.378
Other body parts	7.90			7.47		8.42	
Nose^c^	7.75		0.178	6.87	0.048	7.47	0.024
Face, other parts	8.58			7.88		8.54	
Number of treated BCC							
1	8.35		0.476	7.66	0.898	8.25	0.993
≥2	8.15			7.41		8.20	
Histological subtype^b^							
Nodular	8.37		0.859	7.78	0.868	8.39	0.257
Superficial	8.40			7.41		8.49	
Infiltrating	8.03			7.32		7.75	
Wound closure^b,d^							
Linear closure	8.45		0.927	8.04	0.319	8.83	0.036
Advancement or rotation flap	8.07			7.29		7.76	
Transposition flap	8.20			6.76		8.33	
Skin graft	8.55			7.97		8.33	
Complications							
Yes	7.46		0.007	6.57	0.020	7.05	0.002
No	8.45			7.78		8.49	
Pain							
Yes	7.4		0.004	6.34	<0.001	7.39	0.002
No	8.67			8.12		8.62	
Wound healing							
≤2 weeks	9.40		0.003	9.59	<0.001	9.23	0.032
>2-6 weeks	8.45			7.74		8.31	
>6 weeks	7.56			6.51		7.77	

**Table 5:** Multivariate regression models assessing the impact of patient, tumor, and treatment characteristics on satisfaction at T0. β represents the standardized regression coefficient. For metric variables (age and general health state), a positive β value indicates rising treatment satisfaction with an increase of the respective variable. For all other variables, a positive β-value signifies greater satisfaction compared to the reference group. Significant findings are highlighted in bold. Reference groups: ^a^ male, ^b^ single, ^c^ apprenticeship, ^d^ ≤3 comorbidities, ^e^ other tumor localization, ^f^ linear closure after ellipse excision, ^g^ no complication, ^h^ no pain.

Characteristics	Global satisfaction		Cosmetic satisfaction		Functional satisfaction	
	β	p	β	p	β	p
Female^a^	-0.006	0.941	-0.210	0.027	-0.120	0.176
Age	0.033	0.696	0.105	0.251	0.012	0.889
Partner^b^	-0.095	0.272	-0.007	0.938	-0.014	0.870
University degree^c^	0.104	0.217	0.078	0.386	0.135	0.114
General health state	0.344	<0.001	0.208	0.023	0.305	0.001
>3 comorbidities^d^	-0.115	0.213	-0.134	0.174	-0.015	0.869
Head/neck localization^e^	-0.079	0.410	-0.040	0.710	-0.006	0.955
Advancement or rotation flap^f^	0.112	0.331	0.204	0.096	0.051	0.667
Transposition flap^f^	-0.001	0.988	0.112	0.284	-0.032	0.753
Graft^f^	0.054	0.560	0.135	0.177	0.126	0.183
Complications^g^	-0.273	0.002	-0.324	0.001	-0.155	0.090
Pain^h^	-0.128	0.151	-0.109	0.252	-0.238	0.010

At T0, no significant differences were found with respect to age and marital status (Table 3). However, older participants (>75 years) and participants living with a partner reported significantly better satisfaction with functional results after three months (Table 4). The association with age was confirmed in regression models (ß = 0.204, p = 0.029; Table 6).

Patients with ≤3 comorbidities reported higher global satisfaction than those with >3 concomitant diseases at T0 but not at T1 (Tables 3, 4). A poor health state was associated with lower global and functional satisfaction at both time points (Tables 3, 4). This finding was substantiated in multivariate regression models which additionally showed a relation between poor health state and lower satisfaction with the cosmetic outcome at T0 (ß = 0.208, p = 0.023; Table 5).

Participants with BCC on the nose stated lower global and cosmetic satisfaction compared to patients with BCC on other parts of the face at suture removal (Table 3), and lower cosmetic and functional satisfaction after three months (Table 4), findings that were partly corroborated by multivariate regression analysis (data not shown).

Linear wound closure after ellipse excision led to better global satisfaction than local flaps and skin grafts after three months (Table 6). Postoperative complications and pain were associated with lower satisfaction in all categories and at both time points (Tables 3-6), whereas a wound healing period of ≤2 weeks was related to higher global, functional, and cosmetic satisfaction (Table 4). Concordantly, regression models revealed higher satisfaction with cosmetic outcomes upon short wound healing (Table 6).

**Table 6:** Multivariate regression models assessing the impact of patient, tumor, and treatment characteristics on satisfaction at T1. β represents the standardized regression coefficient. For metric variables (age and general health state), a positive β value indicates rising treatment satisfaction with increase of the respective variable. For all other variables, a positive β-value signifies greater satisfaction compared to the reference group. Significant findings are highlighted in bold. Reference groups: ^a^ male, ^b^ single, ^c^ apprenticeship, ^d^ ≤3 comorbidities, ^e^ other tumor localization, ^f^ linear closure after ellipse excision, ^g^ no complication, ^h^ no pain, ^i^ wound healing ≤2 weeks.

Characteristics	Global satisfaction		Cosmetic satisfaction		Functional satisfaction	
	β	p	β	p	β	p
Female^a^	0.031	0.738	-0.087	0.373	0.006	0.953
Age	0.087	0.318	0.164	0.076	0.204	0.029
Partner^b^	0.116	0.197	-0.069	0.467	0.086	0.364
University degree^c^	0.039	0.651	-0.039	0.665	0.096	0.297
General health state	0.064	0.473	0.117	0.216	0.237	0.014
>3 comorbidities^d^	-0.060	0.530	-0.085	0.395	0.131	0.203
Head/neck localization^e^	0.206	0.067	0.065	0.582	-0.025	0.833
Advancement or rotation flap^f^	-0.434	0.001	-0.065	0.629	-0.291	0.035
Transposition flap^f^	-0.214	0.033	-0.177	0.092	-0.131	0.212
Graft^f^	-0.260	0.007	0.056	0.577	-0.047	0.644
Complications^g^	-0.254	0.009	-0.104	0.303	-0.249	0.015
Pain^h^	0.003	0.976	-0.183	0.061	-0.259	0.010
Wound healing >2-6 weeks^i^	-0.141	0.340	-0.405	0.010	-0.179	0.238
Wound healing >6 weeks^i^	-0.278	0.075	-0.543	0.001	-0.061	0.704

The number of skin tumors treated simultaneously (1 vs. ≥2 BCC) and the histological subtype of the predominantly treated BCC had no significant impact on treatment satisfaction (Tables 3, 4).

Well in line with patient-reported outcomes, physician’s satisfaction with the cosmetic and functional outcome was high at T0 (mean: 8.4 and 8.6). Patients’ and physicians’ satisfaction were intercorrelated (Spearman’s correlation coefficient ρ = 0.321, p < 0.001 for cosmetic satisfaction; ρ = 0.273, p = 0.001 for functional satisfaction).

## Discussion

Our participants reported overall high global, cosmetic, and functional satisfaction, which was significantly influenced by various patient- and tumor-related factors, as well as by the reconstruction technique and by postoperative complications.

Impact of patient characteristics

Our finding that females were more critical of the cosmetic outcome is likely due to the fact that women generally place more importance on their appearance, not least because of societal expectations [25]. In a recent study on the impact of gender on the treatment of BCC in the head/neck region, women tended to rate their scars more critically than males, although physicians’ assessments of the scar quality did not differ significantly [26]. Moreover, women had higher health-related quality of life impairment due to their BCC than men [26].

Satisfaction with functional outcomes was higher in older participants compared to younger ones. This disparity may be attributed to the decreasing skin turgor of older patients, facilitating tension-free wound closure and consequently leading to better functional results. In addition, incisions may be placed in pre-existing folds, resulting in less impairing scars. Furthermore, it seems plausible that expectations regarding the functional outcome decrease with increasing age. In line with our findings, Ofaiche et al. reported better outcomes of BCC surgery in aged skin based on the Patient and Observer Scar Assessment Scale (POSAS) [22]. By contrast, in a study by Asgari et al., older patients were less satisfied with conventional and micrographically controlled excisions than younger ones [21]. However, this study also showed a positive correlation between general health state and treatment satisfaction, concordant with our data [21].

Impact of treatment characteristics

The most common technique used for wound closure in our study was local flaps (60.7%), followed by primary linear closure after ellipse excision (27.3%) and skin grafts (6%). In a study by Kara et al. on the surgical management of BCC in the head/neck area, 44% of the defects were repaired by local flaps, 37.8% with linear closure, and 17.6% with grafts [27]. Our center receives referrals of multimorbid and aged patients with large and difficult-to-treat BCC in challenging localizations. Therefore, reconstruction could be managed by linear closure only in a minority of cases. Cosmetic satisfaction was particularly high with this method, which is simple and entails a low risk for complications but has its limitations in large defects and challenging facial localizations such as the nose. The highest satisfaction with linear closure was also demonstrated in a study by Rustemeyer et al. on 205 patients with facial BCC, in which 92.6% of the participants with linear closure and 88.4% with local flaps, but only 66.4% with full-thickness skin graft and 54% with split-thickness skin graft rated the cosmetic outcome as good or very good [28]. Similarly, functional outcome was rated best after linear closure according to our results and the study by Rustemeyer et al. [28]. Our data underline that this safe and simple technique should be used preferentially when suitable.

Our study did not show significant differences in satisfaction with local flaps compared to skin grafts, possibly because skin grafts were predominantly used for defect coverage on the scalp and not in the central face. Comparing patient satisfaction with local flaps and skin grafts in the face, Lee et al. found higher cosmetic satisfaction with flaps [29].

Our data demonstrate that complications and pain are major contributors to dissatisfaction. Pain often indicates complications. Complications may, in turn, lead to poor cosmetic and functional outcomes. In addition, complications are associated with longer periods of wound healing. It is therefore self-explanatory that patients who had suffered pain and complications were less satisfied with the cosmetic and functional results. The relatively high rate of complications observed in our study may be explained by the facts that (1) wound irritations and mild healing disturbances were counted as complications; (2) the patients had large BCC in challenging localizations; (3) the study cohort contained many geriatric, multimorbid, and immunosuppressed patients and patients on anticoagulation; and (4) several participants underwent surgery of more than one tumor in the same session. The vast majority of complications were mild and reversible within a few weeks.

Limitations

The main limitations of our study are the monocentric setting and the limited number of participants. The cohort comprised predominantly patients with BCC in the head/neck region and with large tumors and is therefore representative of dermatological hospitals and centers specialized in skin cancer surgery, but not of dermatological practices. Clearly, our study would gain relevance if it was expanded to multiple centers and to larger and more diverse patient cohorts.

The patients were recruited for study participation after surgery, and the clinical size of the BCC was not exactly recorded preoperatively in some cases. Many patients presented to our department with BCC that had been previously biopsied, partly excised, or excised R1 in a dermatological practice before referral to our clinic. This precluded consistent and reliable documentation of the original tumor size, which clearly impacts the surgical approach, reconstruction technique, risk of complications and wound healing disturbance, and, thereby, patient satisfaction.

Treatment satisfaction was influenced more by the general health status than by the number of comorbidities, which were highly heterogeneous. In order to capture the severity of comorbidities and their impact on mortality, it will be expedient to record the Charlson Comorbidity Index in addition to the number and kind of comorbidities in future studies. Moreover, it would be interesting to investigate the impact of single comorbidities on patients’ satisfaction with surgery of BCC in a larger cohort.

In subgroup analyses of treatment satisfaction according to the type of wound closure and in multivariate regression models, satisfaction with special types of flaps that were used only rarely, such as S, H, V-to-T, and W-to-Y flaps, and satisfaction with secondary intention healing could not be analyzed separately due to the small numbers of cases. Moreover, for the reconstruction techniques used more commonly such as linear closure, advancement and rotation flap, transposition flap, and skin graft, it would be desirable to explore differences in satisfaction and outcomes based on the tumor localization and the complexity of the reconstruction in greater detail. However, this would require a larger study cohort and higher numbers of BCC in each localization.

The follow-up examination was performed as early as three months after surgery. It is conceivable that satisfaction with the cosmetic and functional outcome would be higher after a longer period. Expanding the follow-up would strengthen our findings and allow for a better evaluation of long-term satisfaction and outcomes including long-term cosmetic and functional results and recurrence rates. On the other hand, a follow-up at a later time period would involve a higher risk of recall bias and a higher dropout rate in the aged collective. The follow-up assessment was performed by mail, and patient-reported outcomes were not verified by clinical examination at this time point.

The fact that the study questionnaire was in German language and that patients without German language skills were excluded from study participation limits the generalizability of our results to more diverse populations. Among the patients who receive BCC surgery in our department, more than 90% speak German, even if not all of them are native speakers. In case of difficulties with understanding parts of the questionnaire due to language barriers or other issues, assistance was offered by the study team.

Another limitation is that we did not assess psychological aspects such as anxiety or body image concerns, which may significantly influence patients' satisfaction. It will be important and interesting to consider these aspects in future studies.

Finally, we used VAS instead of more specific scores such as the POSAS to assess treatment satisfaction.

## Conclusions

Our study demonstrated overall high satisfaction with BCC surgery and identified several important influencing factors. Satisfaction was highest with linear closure, a safe and simple approach, but also varied significantly depending on gender, age, and general health status. All of these features have to be taken into account along with the tumor size and localization when choosing the optimal approach for each patient. Anticipated results and impact factors on patient-perceived outcomes should be discussed in detail during informed decision-making in order to reconcile patients' expectations with practicability.

## Appendices

Appendix A

The patient questionnaire T0 (German language) can be accessed via https://docs.google.com/document/d/e/2PACX-1vR5Pn4QrKaiNJTVXBLlnUJ6pi-6wv1tus_HWPuthFKOlZIzJGAaUBaofX0vfjY9wu7vZKO9Cpm8QXqc/pub

The patient questionnaire T0 (English language) can be accessed via https://docs.google.com/document/d/e/2PACX-1vTgFvbNg3lqgefJp8rqWsf1TOhoMmBhbv9L3i9Kldc_Fud72razZotFvLL21LAKQiPduHug2qDTimmq/pub

The patient questionnaire T1 (German language) can be accessed via https://docs.google.com/document/d/e/2PACX-1vTO-gCi6zJzSJGewM1owBUjSc05jJObGJa_1utywOrQsM_D0hD9G_HW0h-LZ0-j8UBwkZsBMgLISBzt/pub

The patient questionnaire T1 (English language) can be accessed via https://docs.google.com/document/d/e/2PACX-1vQWZ6w9K3F4fzrIoReZHF94vB6-lRkZ5VoPm7yYDtaIodqHX8-pwWRCmQfbHPlGZPdWuDfHwXAaPZ04/pub

Appendix B

**Table 7:** Comorbidities. ^a^ Other than skin cancer.

Comorbidity	n (%)
Arterial hypertension	106 (70.7%)
Cardiovascular disease	78 (52.0%)
Diabetes mellitus	41 (27.3%)
Other metabolic disease	52 (34.7%)
Cancer^a^	23 (15.3%)
Musculoskeletal disease	48 (32.0%)
Immunosuppression	3 (2.0%)
Hematological disease	12 (8.0%)
Thyroid disease	28 (18.7)
Renal disease	23 (15.3%)
Pulmonary disease	9 (6.0%)
Autoimmune disease	2 (1.3%)
Neurological disease	16 (10.7%)
Psychiatric disease	12 (8.0%)
Chronic pain	7 (4.7%)
Other	69 (46.0%)

Appendix C

**Table 8:** Anticoagulation. ^a^ 44.0% of the total cohort (n = 150 patients) received anticoagulation. ^b^ For the calculation of the percentages of specific anticoagulants, the number of patients with anticoagulation (n = 66) was set to 100%.

Anticoagulation	n (%)
Total	66 (44.0%)^a^
Platelet aggregation inhibitor^b^	39 (59.1%)
Vitamin K antagonist^b^	4 (6.1%)
Factor Xa inhibitor^b^	19 (28.8%)
Thrombin inhibitor^b^	2 (3.0%)
Not specified^b^	2 (3.0%)

Appendix D

**Table 9:** Complications. ^a^ Complications reported by patients and validated by physicians.

Complication^a^	n (%)
Irritated wound	22 (14.0%)
Wound infection	9 (6.0%)
Wound dehiscence	9 (6.0%)
Disturbed wound healing	6 (4.0%)
Nerve injury	3 (2.0%)
Secondary bleeding	1 (0.8%)
Allergic reaction	1 (0.8%)
